# The new seriniquinone glycoside by biological transformation using the deep sea-derived bacterium *Bacillus licheniformis* KDM612

**DOI:** 10.1038/s41429-024-00729-z

**Published:** 2024-05-21

**Authors:** Ryota Okamura, Katsuki Kikuchi, Akito Taniguchi, Kenichiro Nagai, Reiko Seki, Satoshi Ohte, Taichi Ohshiro, Masashi Ando, Teruyoshi Tanaka, Takashi Fukuda

**Affiliations:** 1 Department of Fisheries, Faculty of Agriculture, Kindai University, Nara, Japan; 2 Microbial Chemistry and Medicinal Research Laboratories, Graduate School of Pharmaceutical Sciences, Kitasato University, Tokyo, Japan; 3 Agricultural Technology and Innovation Research Institute, Kindai University, Nara, Japan

**Keywords:** Phenotypic screening, Cancer screening

## Abstract

Seriniquinone was isolated as a melanoma-selective anti-cancer agent from a culture broth of the marine-derived bacterium *Serinicoccus marinus* CNJ927 in 2014. It targets the unique small protein, dermcidin, which affects the drug resistance of cancer cells. Due to its significant activity against cancer cells, particularly melanoma, and its unique target, seriniquinone has been developed as a new pharmacophore. However, it has the disadvantage of poor solubility in drug discovery research, which needs to be resolved. A new seriniquinone glycoside (**1**) was synthesized by the biological transformation of seriniquinone using the deep sea-derived bacterium *Bacillus licheniformis* KDM612. Compound **1** exhibited selective anti-cancer activity against melanoma, similar to seriniquinone, and was 50-fold more soluble in DMSO than seriniquinone.

## Introduction

In recent years, cancer treatment has made dramatic progress with the advent of molecular targeted therapies, immunotherapy, and genomic medicine, in addition to conventional chemotherapy [1]. However, each of these therapies has advantages and disadvantages, and there are still many challenges that need to be overcome. In addition, drug resistance in cancer has recently become an issue [2]. Many mechanisms of drug resistance have been reported, including the enhancement of drug efflux pumps, the activation of anti-apoptotic signals, a reduction in drug-target affinity, and the mutation of oncogenes; however, many remain unknown and no fundamental solution has been found [3–5]. Therefore, the provision of new target molecules and the discovery of lead compounds that overcome drug resistance are urgently desired. In 2012, the protein dermcidin (DCD) was shown to induce drug resistance to anti-cancer drugs and play a role in cancer metastasis [6]. DCD is an anti-microbial peptide that was discovered in human sweat in 2001 [7]. The expression of DCD has been detected in skin, breast, ovarian, and lung cancers [8]. Furthermore, its knockdown was found to inhibit the growth and tumorigenesis of breast and lung cancers [9]. In melanoma cells, the most drug-resistant type of skin cancer, oxidative stress has been shown to induce the RNA expression of DCD [10]. Collectively, these findings clearly demonstrate the involvement of DCD in the survival and drug resistance of various cancers.

**Fig. 1:**
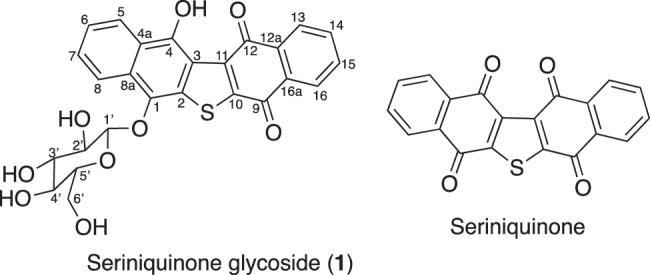
Structures of compound **1** and seriniquinone

Against this background, seriniquinone was discovered in a culture of the marine bacterium *Serinicoccus marinus* CNJ927 [11]. This compound exhibited selective and potent anti-cancer activity against melanoma cells with high drug resistance. A mechanism of action analysis revealed that seriniquinone directly bound to DCD and regulated its function [11]. This finding indicates that seriniquinone may lead to a breakthrough drug to overcome drug resistance in cancer. However, its solubility is poor and various seriniquinone derivatives need to be created for drug discovery [12–14]. Under this concept, a dihydronaphthothiophene derivative (**2**) was identified as a derivative of seriniquinone through its biological transformation using marine microorganisms [15]. Compound **2** was 100-fold more soluble in DMSO than seriniquinone, but also showed a decrease in activity. We continued our research on seriniquinone derivatives by biological transformation and found strain KDM612 of a deep sea-derived bacterium converted seriniquinone to the novel seriniquinone glycoside (**1**) (Fig. 1). We herein describe methods for the transformation, isolation, structural elucidation, and to assess cytotoxicity of compound **1**.

## Results and discussion

### Selection of strain KDM612

**Fig. 2:**
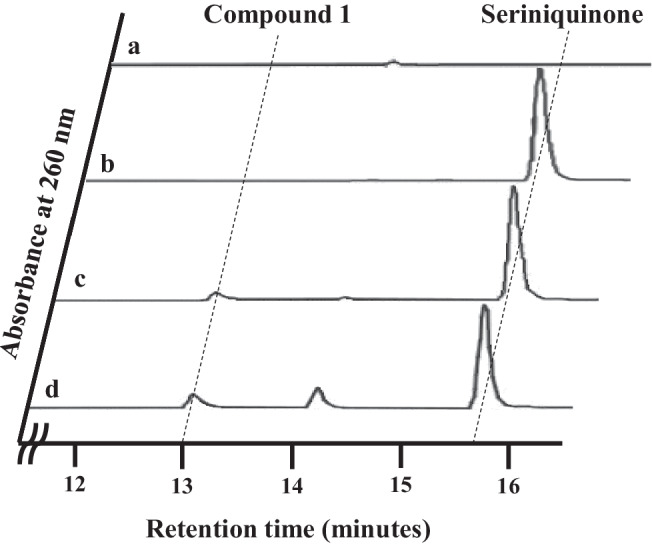
An analysis of production of compound **1** by HPLC; column, LaChromUltra C18 (2 × 75 mm); flow rate, 0.25 ml min^-1^; mobile phase, **a** 15-min linear gradient from 5% CH_3_CN to 95% CH_3_CN and 5-min 95% CH_3_CN with 0.05% H_3_PO_4_; detection, UV at 260 nm. a 7 days-culture broth of strain KDM612 without seriniquinone. **b** 0 day-culture broth of strain KDM612 with seriniquinone. **c** 3 days-culture broth of strain KDM612 with seriniquinone. **d** 7 days-culture broth of strain KDM612 with seriniquinone

As a result of screening 838 marine-derived microorganisms, strain KDM612 was selected. When strain KDM612 was cultured with seriniquinone added to the medium, the peak of seriniquinone (R.T. 15.7 min) decreased gradually and a new peak with retention time of 12.9 min appeared on the HPLC result (Fig. 2). In the 7 days-culture broth, the new peak became maximum. On the other hands, this new peak (R.T. 12.9 min) did not detect without the addition of seriniquinone.

### Collection and identification of strain KDM612

**Fig. 3:**
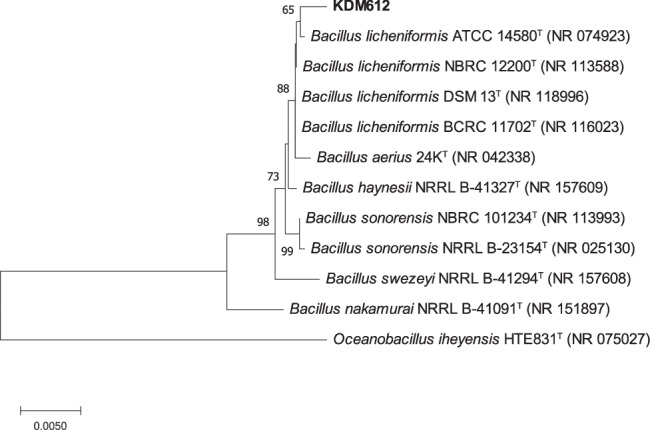
Neighbor-joining tree of 16 S rRNA gene sequence of strain KDM612. Bootstrap values above 50% are shown next to the branches. *Oceanobacillus iheyensis* HTE831^T^ (NR_075027) is used as an outgroup. The scale bar represents 0.5% estimated divergence

Strain KDM612 was isolated from a marine creature collected at a depth of 300 m in Suruga Bay, Shizuoka, Japan in 2022. This strain was cultured on inorganic salt starch agar and had a pale yellow, feather-like morphology (see Fig. S1). Species identification was conducted through direct colony PCR using 16 S rRNA gene primers, 27 f forward primer (5’-AGR GTT TGA TYM TGG CTC AG -3’) and 1492r reverse primer (5’-GGH TAC CTT GTT ACG ACT T-3’), in a 20 μl reaction mixture. The reaction mixture contained a final concentration of 1 × PCR buffer, 400 µM of each dNTP, 500 nM of each primer, and 0.02 U/μl KOD FX Neo (TOYOBO, Osaka, Japan). Bidirectional sequencing was performed using an ABI 3130xl sequencer (Applied Biosystems, Foster City, CA, USA) after sequencing reaction with a BigDye^TM^ Terminator v3.1 Cycle Sequencing Kit (Applied Biosystems, Foster City, CA, USA) using the same primers as mentioned above. The obtained sequence was compared to known sequences in the National Center for Biotechnology Information (NCBI) nucleotide database using the Basic Local Alignment Search Tool (BLAST). Phylogenetic relationships were determined by generating multiple alignments using CLUSTAL W, and the phylogenetic tree was constructed using the neighbor-joining method with 1000 bootstrap replicates, using MEGA version X [16]. Strain KDM612 shared 99.5% identity with *Bacillus licheniformis* (Fig. 3). The nucleotide sequence has been deposited in the DNA Data Bank of Japan (DDBJ) with the accession number LC782819.

### Transformation and isolation of 1

Strain KDM612 was inoculated into a 100-ml Erlenmeyer flask containing 25 ml of seed medium (1.0% soluble starch, 0.4% yeast extract, and 0.2% peptone in natural sea water). The flask was shaken on a rotary shaker at 27 °C for 7 days. The seed culture (25 ml) was transferred into a 2-l Erlenmeyer flask containing 500 ml of production medium (1.0% soluble starch, 0.4% yeast extract, 0.2% peptone, 0.1% CaCO_3_, 0.004% Fe_2_(SO_4_)_3_・nH_2_O, and 0.01% KBr in natural sea water). After shaking at 180 rpm at 27 °C for 7 days, seriniquinone (25 mg) was added to the culture and a conversion culture was performed under the same conditions for 7 days.

The culture broth (1.0 l) was added to Amberlite-XAD7 (20 ml) and shaken for 2 h. After filtration, filtrate residue was extracted with acetone overnight. The acetone layer was evaporated to the 10 ml volume. This material was applied to InertSep^TM^C18 (10 g/60 ml, GL Sciences, Shinjuku, Japan), and eluted stepwise with 0, 30, 60, 80 and 100% aqueous CH_3_CN, 100 ml each. The 60% aqueous CH_3_CN fraction was concentrated to yield a green material (10.0 mg). This material was further purified by reverse phase HPLC using a C-18 packed column (20 × 250 mm; PEGASIL ODS sp100, Senshu Scientific Co., Tokyo, Japan) under the following conditions: solvent, 50% aqueous CH_3_CN in 30 min at a flow rate of 8.0 ml min^−1^ with UV detection at 250 nm. Under these conditions, compound **1** was eluted as a peak with a retention time of 25.0 min. This peak was collected and concentrated to yield pure **1** (0.8 mg). The 100% aqueous CH_3_CN fraction was concentrated to yield a green material (43.3 mg). This material was further purified by the same strategy as the 60% aqueous CH_3_CN fraction, compound **1** was purified to yield 3.2 mg.

### Structural elucidation of 1

The physicochemical properties of compound **1** are summarized in Table 1. It showed absorption maxima at 221, 258, 288 and 337 nm in the UV spectrum. Absorption at approximately 3380 and 1648 cm^−1^ in IR spectra suggested the presence of hydroxyl and carbonyl groups.

**Table 1:** Physicochemical properties of **1**

	**1**
Appearance	Green amorphous powder
Molecular formula	C_26_H_20_O_9_S
Molecular weight	508
HR ESI MS *m/z*	[M + H]^+^
Calcd	509.0906 (for C_26_H_21_O_9_S)
Found	509.0897
UV λ ${\rm max}^{\rm MeOH}$ nm	337, 288, 258, 221
IR ν ${\rm max}^{\rm KBr}$ cm^−1^	3380, 2917, 1648, 1580, 1506

**Fig. 4:**
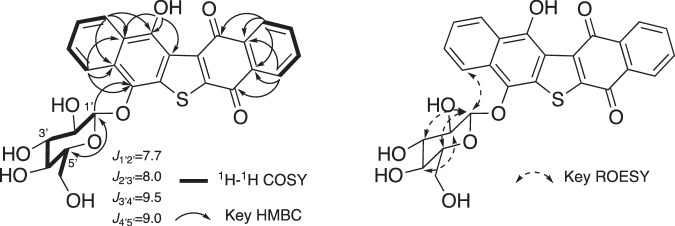
^1^H-^1^H COSY and key HMBC correlations of compound **1**

Compound **1** was obtained as green amorphous powder. The molecular formula for **1** was elucidated as C_26_H_20_O_9_S ([M + H]^+^ *m/z* 509.0897, calcd [M + H]^+^ 509.0906) based on high-resolution ESI-MS measurements, indicating that **1** contained 17 degrees of unsaturation (Table 1). ^1^H and ^13^C NMR data (in pyridine-*d*_*5*_) supported the molecular formula (Table 2). The ^13^C NMR spectrum showed 26 resolved signals, which were classified into eight *sp*^2^ methine carbons, ten *sp*^2^ quaternary carbons, two carbonyl carbons (C-9 and C-12), one *sp*^3^ methylene carbon and five *sp*^3^ methine carbons. The ^1^H NMR spectrum of **1** showed eight olefinic methine signals, one methylene signal, five methine signals and one exchangeable signal. The connectivity of all proton and carbon atoms was established by HMQC experiments (Table 2). An analysis of ^1^H-^1^H COSY data allowed two partial structures of 1,2-substituted benzenes and a hexose to be assigned (Fig. 4). An analysis of HMBC spectroscopic data gave further structural information on **1**. (1) Cross-peaks from 4-OH (*δ* 13.03) to C-3 (*δ* 119.6), C-4 (*δ* 150.9) and C-4a (*δ* 124.5), from H-5 (*δ* 8.81) to C-4, C-4a and C-8a (*δ* 129.2) and from H-8 (*δ* 9.30) to C-4a, C-8a and C-1 (*δ* 140.7) supported a hydroxyl naphthoquinone moiety. (2) Cross-peaks from H-1’(*δ* 5.72) to C-5’ (*δ* 79.3) and from H-5’ (*δ* 4.00) to C-1’ (*δ* 108.3) supported a sugar moiety. (3) Cross-peaks from H-13 (*δ* 8.30) to C-12 (*δ* 183.4), C-12a (*δ* 133.4) and C-16a (*δ* 133.8) and from H-16 (*δ* 8.19) to C-12a, C-16a and C-9 (*δ* 179.5) supported a further naphthoquinone moiety. Furthermore, the cross-peak from H-1’ to C-1 supported a hexose moiety being connected to a hydroxyl carbon via oxygen. Collectively, these data and in connection with the known shift values for seriniquinone, revealed the planar structure of **1** as shown in Fig. 1.

**Table 2:** NMR spectroscopic data for **1** in pyridine-*d*_5_

**1**		
position	*δ*_C_^a^	*δ*_H_^b^ mult (*J* in Hz)
1	140.7, C	
2	132.7, C^c^	
3	119.6, C	
4	150.9, C	
4a	124.5, C	
5	124.9, CH	8.81, d (8.6)
6	125.4, CH	7.57, t (7.7)
7	128.7, CH	7.69, t (7.7)
8	122.8, CH	9.30, d (8.6)
8a	129.2, C	
9	179.5, C^d^	
10	134.9, C^c^	
11	137.4, C^c^	
12	183.4, C^d^	
12a	133.4, C^e^	
13	128.8, CH^f^	8.30, d (7.3)
14	135.2, CH^g^	7.73, t (7.2)
15	135.2, CH^g^	7.72, t (7.7)
16	127.1, CH^f^	8.19, d (7.3)
16a	133.8, C^e^	
1'	108.3, CH	5.72, d (7.7)
2'	76.1, CH	4.60, br t (8.0)
3'	78.9, CH	4.39, br t (9.5)
4'	72.1, CH	4.42, br t (9.0)
5'	79.3, CH	4.00, m
6'	63.3, CH_2_	4.50, d (11.0)
		4.40, overlapping
4-OH		13.03, s

Assignments made by interpretation of COSY, HSQC and HMBC NMR data

^a^100 MHz.^b^400 MHz

^c,d,e,f,g^ Exchangable

The relative configuration of a hexose in **1** was elucidated by analysis of the ^1^H-^1^H coupling constants (Table 2). In a sugar (Fig. 4), the large vicinal coupling constants between H-1’ and H-2’ (*δ* 4.60), between H-2’ and H-3’ (*δ* 4.39), between H-3’ and H-4’ (*δ* 4.42), and between H-4’ and H-5’ were 7.7 Hz, 8.0 Hz, 9.5 Hz, and 9.0 Hz, respectively. These results indicated that these protons were axial. These results and the chemical shift of C-1’ revealed that the anomeric configuration of C-1’ was oriented in a β form [17]. Additional Rotating frame Overhause Effect SpectroscopY (ROESY) correlations between H-1’, H-3’ and H-5’ supported to be co-facial and co-axially oriented. Thus, a sugar moiety was identified as a β-glucose. Additionally, the observation of an ROESY correlation between H-8 and H-1’ supported the hydroxyl naphthoquinone and β-glucose moieties being oriented closely.

To investigate the number of hydroxyl group, **1** was acetylated. The observation of 736 mass ([M + NH_4_]^+^) indicated that at least five acetyl methyl groups were incorporated into **1**. This data supported the structure of **1** (see Fig. S10).

### Biological properties

#### Cytotoxic activities of 1

The cytotoxicity of **1** against Malme-3M cells, Jurkat cells and HUVEC cells were measured using the WST-1 or MTT reagent assay, respectively [18–20]. Compound **1** exhibited anti-cancer activity against Malme-3M, Jurkat and HUVEC cells with IC_50_ values of 0.29, 51 and 0.64 μM, respectively (Table 3) (see Fig. S11). On the other hand, compound **1** showed 50-fold better solubility in DMSO than seriniquinone (Table 3).

**Table 3:** Summary of biological activity of compounds

		IC_50_ (μM)		Solubility ^a^
Compound	Malme-3M	Jurkat	HUVEC	
Seriniquinone	0.17	8.7	0.97	0.1
**1**	0.29	51	0.64	5

^a^Solubility = mg / DMSO (1 ml)

## Discussion

In the present study, we screened 838 strains and found that the marine-derived bacterium *B. licheniformis* KDM612 transformed the structure of seriniquinone to that of glycoside (**1**). In this structurally modified product, one quinone moiety of seriniquinone was reduced and one β-glucose was attached. Therefore, solubility increased to 50-fold that of seriniquinone while maintaining the same level of activity as seriniquinone.

**Fig. 5:**
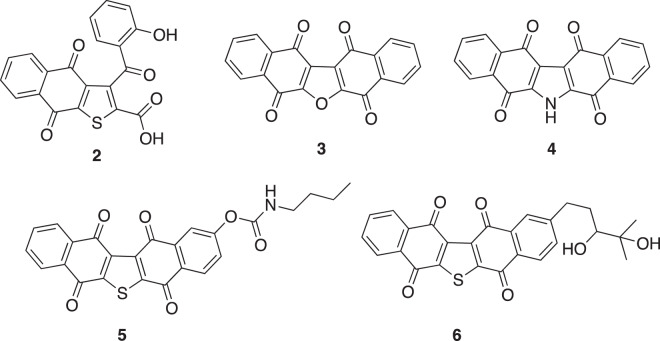
The presentative structures of seriniquinone derivatives (**2**–**6**) that have been reported previously

Several seriniquinone derivatives have been synthesized to date and their structure-activity relationships and improved solubility have been reported (Fig. 5). For example, the anti-cancer activities of compounds **3** and **4** against melanoma cells were reduced, indicating the importance of the central thiophene moiety [13, 21]. The dihydronaphthothiophene derivative (**2**) was biotransformed by *Streptomyces albogriseolus* OM27-12 [15]. Although the solubility of compound **2** was improved, its activity was reduced. This suggested the importance of the pentacyclic structure for anti-cancer activity. The anti-cancer activity, cancer selectivity, and solubility of compounds **5** and **6** were superior to those of seriniquinone [13, 21]. These compounds represent the best candidate therapeutic drugs to date. However, the structures of all reported derivatives had side chains attached to the side benzene ring. Compound **1** has a structure in which a sugar is attached to the hydroquinone moiety, which is completely different from previously reported derivatives. To the best of our knowledge, this is the first study to report a derivative of seriniquinone with a β-glucose and attached side chain in the hydroquinone moiety. The present results provide novel insights into the structure-activity relationship of seriniquinone.

Glycoside compounds are considered to stabilize functional compounds and activate physiological functions. By using enzymes from deep-sea microorganisms, we succeeded in simply and position-selectively adding glucose. Since this reaction is difficult to perform in organic synthesis, it is considered one of the advantages of using enzymes from deep-sea microorganisms. Under the present conditions, only a structure with one molecule of glucose added was confirmed. Under other conditions, such as a longer cultivation, it may be possible to create products with the addition of multiple molecules of glucose. Furthermore, by changing the type of sugar added as a substrate, various types of glycoside compounds may be created. Therefore, biological transformation represents an excellent method for creating new derivatives.

## Experimental section

### General experimental procedures

High-resolution mass spectra were measured with a Xevo G2-XS QTof mass spectrometer equipped with an ESI interface (Waters, Milford, MA, USA). UV and IR spectra were measured with a U-2800 spectrophotometer (HITACHI, Tokyo, Japan) and FT/IR-460 plus spectrometer (JASCO), respectively. NMR spectra were recorded on an ULTRASHIELD PLUS 400 (Bruker, Massachusetts, US). Reverse phase HPLC separation was performed using a Senshu Pak PEGASIL ODS SP100 column (20 × 250 mm) at a flow rate of 8 ml/min with a SHIMADZU LS20AT pump and SHIMADZU LS20AS UV detector (SHIMADZU, Kyoto, Japan).

### Materials

Seriniquinone was synthesized using a previously described method [11].

Soluble starch and Fe_2_(SO_4_)·nH_2_O were purchased from Wako Pure Chemical Industries, Ltd. (Osaka, Japan), yeast extract and peptone from Becton Dickinson (Sparks, MD, USA), and CaCO_3_ and KBr from Kanto Chemical Co., Inc. Iscove’s modified Dulbecco’s medium (IMDM) was purchased from ATCC (Manassas, VA, USA). Roswell Park Memorial Institute (RPMI) 1640 medium was purchased from Sigma-Aldrich (St. Louis, MO, USA). Penicillin (1.0 × 10^4^ units ml^-1^) and streptomycin (1.0 × 10^4 ^mg ml^-1^) solutions were obtained from GIBCO (Grand Island, NY, USA). Fetal bovine serum (FBS) was purchased from BioWest (Riverside, MO, USA). The Premix WST-1 Cell proliferation Assay System was purchased from Takara Bio Inc. (Shiga, Japan).

### Assay for seriniquinone-transformation activity

Marine-derived microorganisms were cultured into a 24-well plate containing 1.5 ml of a production medium with 0.001% seriniquinone. After shaking at 110 rpm at 27 °C for 7 days, methanol (1.0 ml) was added to each culture. This culture broth was analyzed on HPLC under the following conditions: column, LaChromUltra C18 (2 × 75 mm, Hitachi High-Tech Science Co., Tokyo, Japan); flow rate, 0.25 ml min^-1^; mobile phase, a 15-min linear gradient from 5% CH_3_CN to 95% CH_3_CN and 95% CH_3_CN with 0.05% H_3_PO_4_; detection, UV at 260 nm. Under these conditions, seriniquinone was eluted as a peak with a retention time of 15.7 min. In the screening, the sample was selected in which the peak of 15.7 min decreased and another new peak appeared.

### Cell culture

Malme-3M cells were cultured in IMDM medium supplemented with 10% FBS, 100 units ml^-1^ penicillin, and 100 μg ml^-1^ streptomycin at 37 °C in a humidified atmosphere of 5% CO_2_.

HUVEC cells were cultured using the same method, except with DMEM medium.

Jurkat cells were cultured using the same method, except with RPMI 1640 medium.

### Cell viability assay

The effects of compounds on the viability of Malme-3M and Jurkat cells were evaluated using WST-1 (4-[3-(4-iodophenyl)-2-(4-nitrophenyl)-2H-5-tetrazolio]-1,3-benzene disulfonate) reagent. Malme-3M cells (1.0 × 10^4^ cells in 100 μl) were prepared and cultured in a 96-well microplate at 37 °C for 24 h. After the removal of medium, each sample dissolved in 100 μl medium was added and incubated at 37 °C for 72 h. After being incubated, cells were treated with 10 μl WST-1 reagent and then incubated at 37 °C for 30 min. The absorbance of each well was read at 480 nm with MICROPLATE READER SH-9000.The inhibition of cell growth was defined as (absorbance-sample/absorbance-control) × 100. The IC_50_ value was defined as the sample concentration that inhibited cell growth by 50%. Jurkat cells were investigated using a previously described method [19].

The effects of compounds on the viability of HUVEC cells was measured by the colorimetric assay using 3-(4,5-dimethylthiazol-2-yl)2,5-diphenyl tetrazolium (MTT) [20]. In brief, HUVEC cells (1x10^4^ cells in 100 μl) were added to each well of a 96-well microplate. After recovery overnight, the cells were incubated in the absence or presence of compounds (1 µl MeOH solution) for 72 h at 37 °C in 5% CO_2_. After being incubated, cells were treated with 10 μl MTT solution (5.5 mg ml^-1^ in PBS solution) and were then incubated at 37 °C in 5% CO_2_ for 3 h. A 90-μl aliquot of the lysis solution (40% N, N-dimethylformamide, 2.0% CH_3_COOH, 20% SDS and 0.03 M HCl) was added to each well, and the plates were incubated for 15 min at room temperature. The absorbance at 570 nm of each well was measured with iMark microplate reader. The inhibition of cell growth was defined as (absorbance-drug/absorbance-control) × 100. The IC_50_ value was defined as a sample concentration that causes 50% inhibition of cell growth.

### Acetylation of 1

Compound **1** (1.0 mg) was dissolved in dry pyridine (500 μl) and dry acetic anhydride (500 μl) was added at 50 °C for 5 h. After the addition of methanol (500 μl), the reaction mixture was evaporated in vacuo to obtain acetylated product (1.2 mg). This material was used for MS measurement.

### Supplementary information

Supporting Information

## References

[1] Debela DT, et al. New approaches and procedures for cancer treatment: Current perspectives SAGE. Open Med. 2021;9:20503121211034366. 10.1177/20503121211034366 PMC8366192 34408877

[2] Vasan N, et al. A view on drug resistance in cancer. Nature. 2019;575:299–309. 31723286 10.1038/s41586-019-1730-1 PMC8008476

[3] Haider T, et al. Drug resistance in cancer: mechanisms and tackling strategies. Pharmacol Rep. 2020;72:1125–51. 32700248 10.1007/s43440-020-00138-7

[4] Xue X, Liang XJ. Overcoming drug efflux-based multi drug resistance in cancer with nanotechnology. Chin J Cancer. 2012;31:100–9. 22237039 10.5732/cjc.011.10326 PMC3777470

[5] Wilson TR, et al. Anti-apoptotic mechanisms of drug resistance in cancer. Curr Cancer Drug Targets. 2009;9:307–19. 19442051 10.2174/156800909788166547

[6] Schittek B. The multiple facets of dermcidin in cell survival and host defense. J Innate Immun. 2012;4:349–60. 22455996 10.1159/000336844 PMC6741627

[7] Schittek B, et al. Dermcidin: a novel human antibiotic peptide secreted by sweat glands. Nat Immunol. 2001;2:1133–7. 11694882 10.1038/ni732

[8] Minami Y, et al. Cutaneous mixed tumors: an immunohistochemical study using two antibodies, G-81 and C8/144B. Dermatol Sci. 2004;36:180–2. 10.1016/j.jdermsci.2004.09.002 15541641

[9] Bancovik J, et al. Dermcidin exerts its oncogenic effects in breast cancer via modulation of ERBB signaling. BMC Cancer. 2015;15:70. 25879571 10.1186/s12885-015-1022-6 PMC4353460

[10] Rieg S, et al. Dermcidin is constitutively produced by eccrine sweat glands and is not induced in epidermal cells under inflammatory skin conditions. Br J Dermatol. 2004;151:534–9. 15377337 10.1111/j.1365-2133.2004.06081.x

[11] Trzoss L, et al. Seriniquinone, a selective anticancer agent, induces cell death by autophagocytosis, targeting the cancer-protective protein dermcidin. Proc Natl Acad Sci USA. 2014;111:14687–92. 25271322 10.1073/pnas.1410932111 PMC4205641

[12] Moreira da Silva R, et al. Prediction of seriniquinone-drug interactions by in vitro inhibition of human cytochrome P450 enzymes. Toxicol Vitr. 2020;65:104820. 10.1016/j.tiv.2020.104820 32142840

[13] Hammons JC, et al. Advance of seriniquinone analogues as melanoma agents. ACS Med Chem Lett. 2019;10:186–90. 30783501 10.1021/acsmedchemlett.8b00391 PMC6378664

[14] Nagao H, et al. Comparative analysis of p-terphenylquinone and seriniquinone derivatives as reactive oxygen species-modulating agents. Bioorg Med Chem Lett. 2022;76:128992. 36126897 10.1016/j.bmcl.2022.128992

[15] Ishida K, et al. New dihydronaphthothiophene derivatives by the biological transformation of seriniquinone using marine-derived actinomycete *Streptomyces albogriseolus* OM27-12. J Antibiot. 2022;75:9–15. 10.1038/s41429-021-00484-5 34840331

[16] Kumar S, et al. MEGA X: molecular evolutionary genetics analysis across computing platforms. Mol Biol Evolution. 2018;35:1547–49. 10.1093/molbev/msy096 PMC5967553 29722887

[17] Kankanamge S. et al. Noonindoles G-L: indole diterpene glycosides from the Australian marine-derived fungus Aspergillus noonimiae CMB-M0339. J Nat Prod. 2023;86:508–16. 36662567 10.1021/acs.jnatprod.2c01024

[18] Berridge M, Tan A. Trans-plasma membrane electron transport: a cellular assay for NADH-and NADPH-oxidase based on extracellular, superoxide-mediated reduction of the sulfonated tetrazolium salt WST-1. Protoplasma. 1998;205:74–82. 10.1007/BF01279296

[19] Fukuda T, et al. Isomethoxyneihumicin, a new cytotoxic agent produced by marine *Nocardiopsis alba* KM6-1. J Antibiot. 2017;70:590–94. 10.1038/ja.2016.152 27999443

[20] Mosmann T. Rapid colorimetric assay for cellular growth and survival: application to proliferation and cytotoxicity assays. J Immunol Methods. 1983;65:55–63. 6606682 10.1016/0022-1759(83)90303-4

[21] Hirata AS, et al. Preclinical development of seriniquinones as selective dermcidin modulators for the treatment of melanoma. Mar Drugs. 2022;20:301. 35621952 10.3390/md20050301 PMC9143531

