# Supplementary material for: The new seriniquinone glycoside by biological transformation using the deep sea-derived bacterium Bacillus licheniformis KDM612
Source: J Antibiot (Tokyo). 2024 May 21;77(8):515–21. doi: 10.1038/s41429-024-00729-z (PMC11284089; doi:10.1038/s41429-024-00729-z)

Supporting Information

The new seriniquinone glycoside by the biological transformation using a deep sea-derived bacterium *Bacillus licheniformis* KDM612

Ryota Okamura[1], Katsuki Kikuchi[1], Akito Taniguchi[1], Kenichiro Nagai[2], Reiko Seki[2], Ohte Satoshi[2], Taichi Ohshiro[2], Masashi Ando[1], Teruyoshi Tanaka[1], Takashi Fukuda[1,3]*

[1]Department of Fisheries, Faculty of Agriculture, Kindai University, Nara, Japan

[2]Microbial Chemistry and Medicinal Research Laboratories, Graduate School of Pharmaceutical Sciences, Kitasato University, Tokyo, Japan

[3]Agricultural Technology and Innovation Research Institute, Kindai University, Nara, Japan

* Department of Fisheries, Faculty of Agriculture, Kindai University, 3327-204 Nakamachi, Nara, 631-8505, Japan

E-mail: fukudata@nara.kindai.ac.jp

S1. Image depicting KDM612 strain grown on an inorganic salt starch agar surface.
S2. ^{1}H NMR (Pyridine-d_5, 400 MHz) of compound **1**
S3. ^{13}C NMR (Pyridine-d_5, 100 MHz) of compound **1**
S4. ^{1}H-^{1}H COSY (Pyridine-d_5, 400 MHz) of compound **1**
S5. HSQC (Pyridine-d_5, 400 MHz) of compound **1**
S6. HMBC (Pyridine-d_5, 400 MHz) of compound **1**
S7. ROESY (Pyridine-d_5, 400 MHz) of compound **1**
S8. IR of compound **1**
S9. MS of compound **1**
S10. MS of acetylated compound **1**
S11. Activity of compound **1**

S1. Image depicting KDM612 strain grown on an inorganic salt starch agar surface.

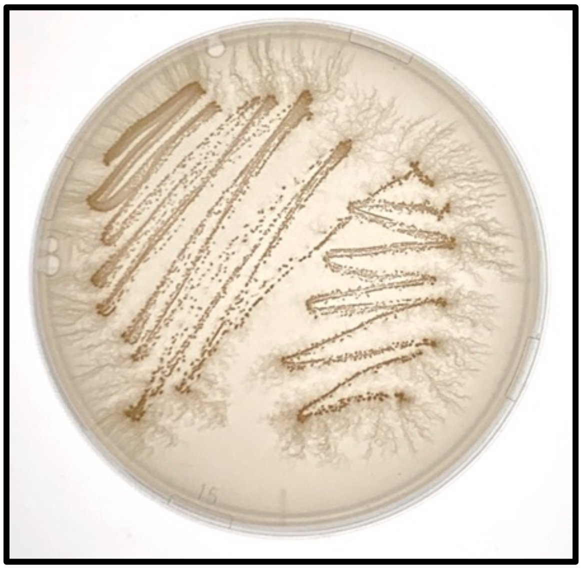

S2. ¹H NMR (Pyridine-d_5, 400 MHz) of compound **1**

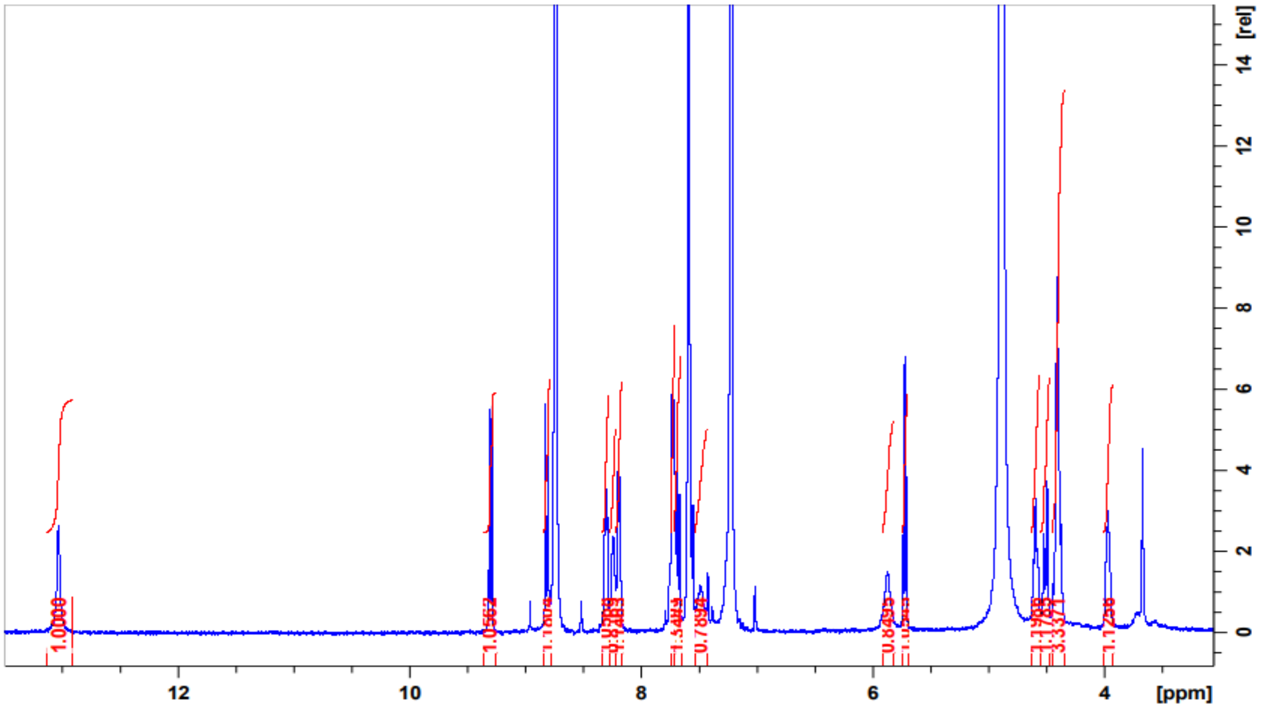

S3. ¹³C NMR (Pyridine-d_5, 100 MHz) of compound **1**

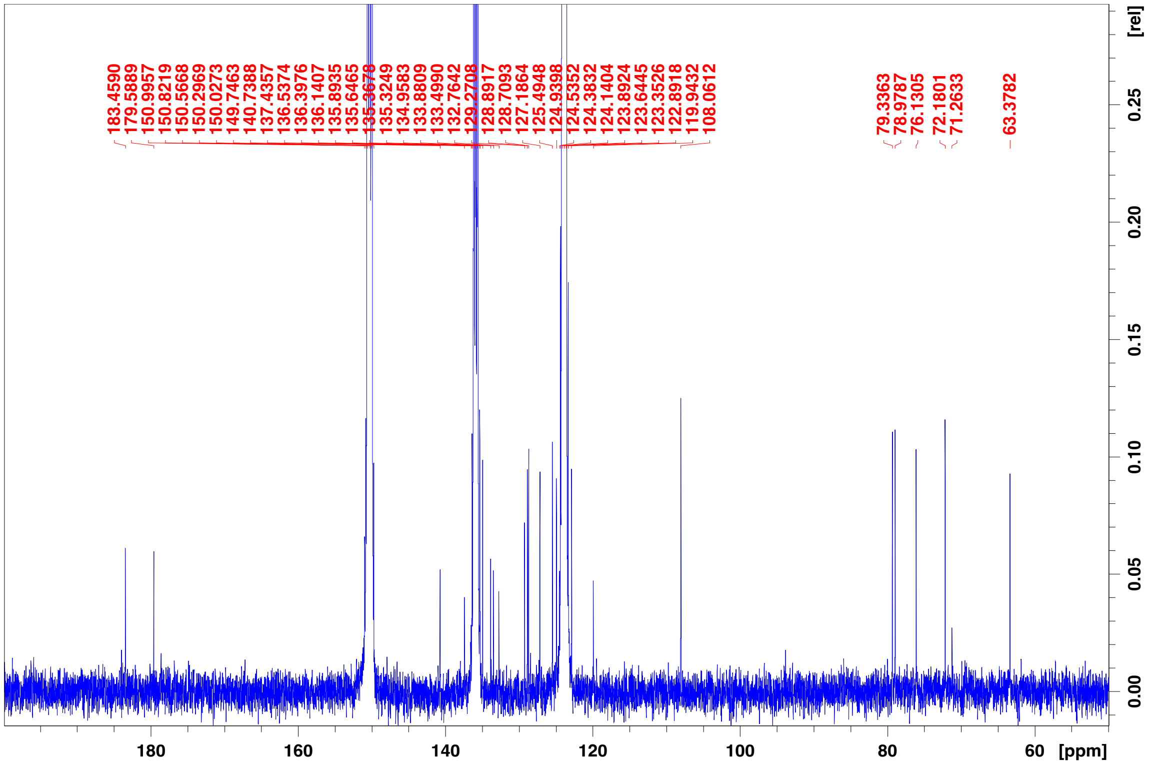

S4. ^{1}H-^{1}H COSY (Pyridine-d_5, 400 MHz) of compound **1**

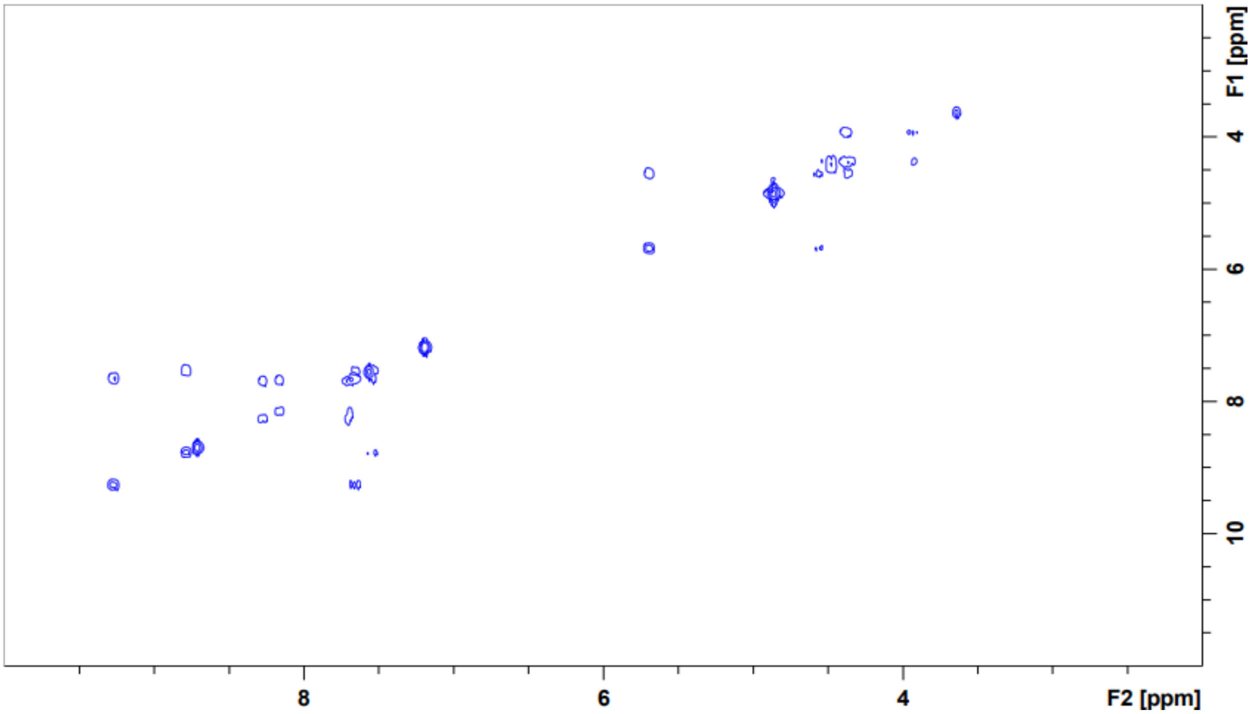

S5. HSQC (Pyridine-d_5, 400 MHz) of compound **1**

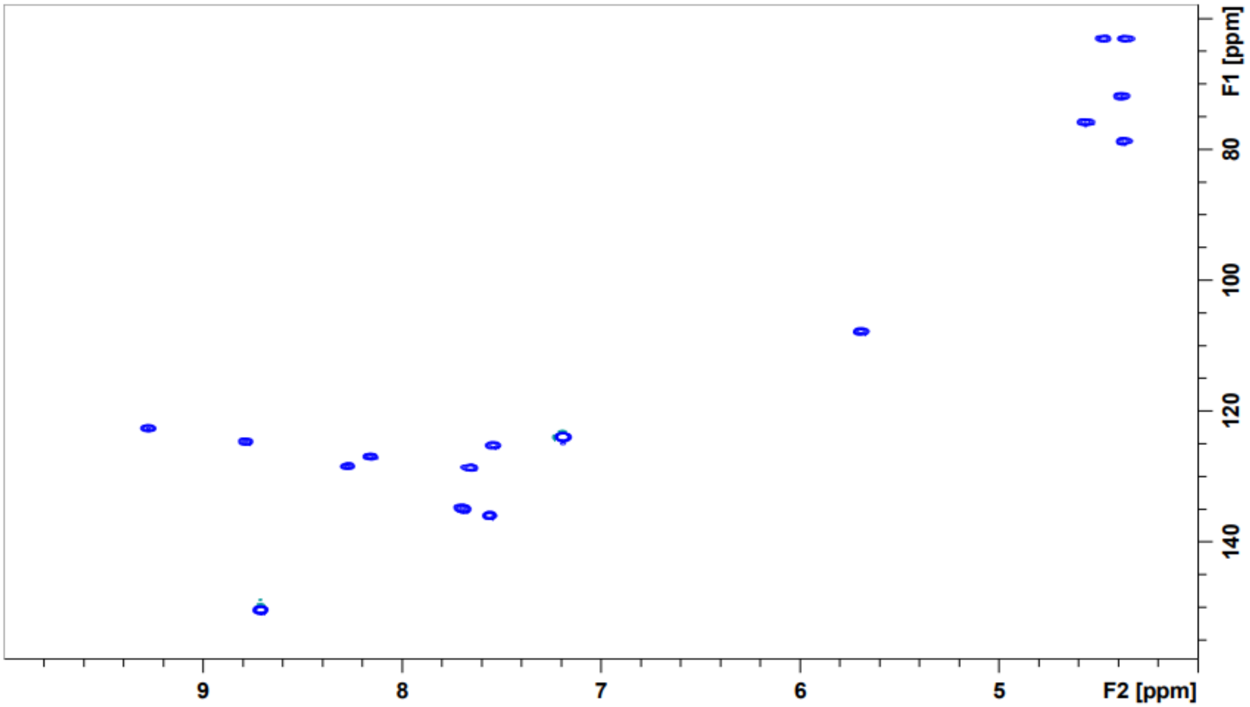

S6. HMBC (Pyridine-d_5, 400 MHz) of compound **1**

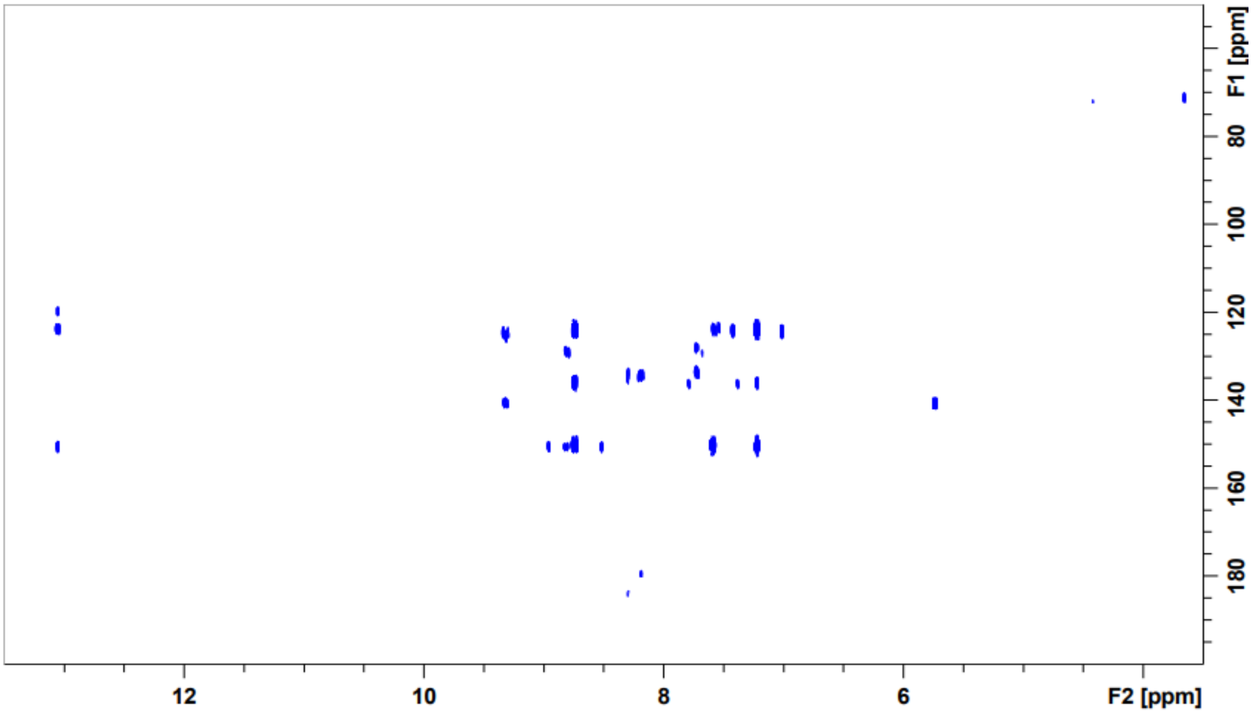

S7. ROESY (Pyridine-d_5, 400 MHz) of compound **1**

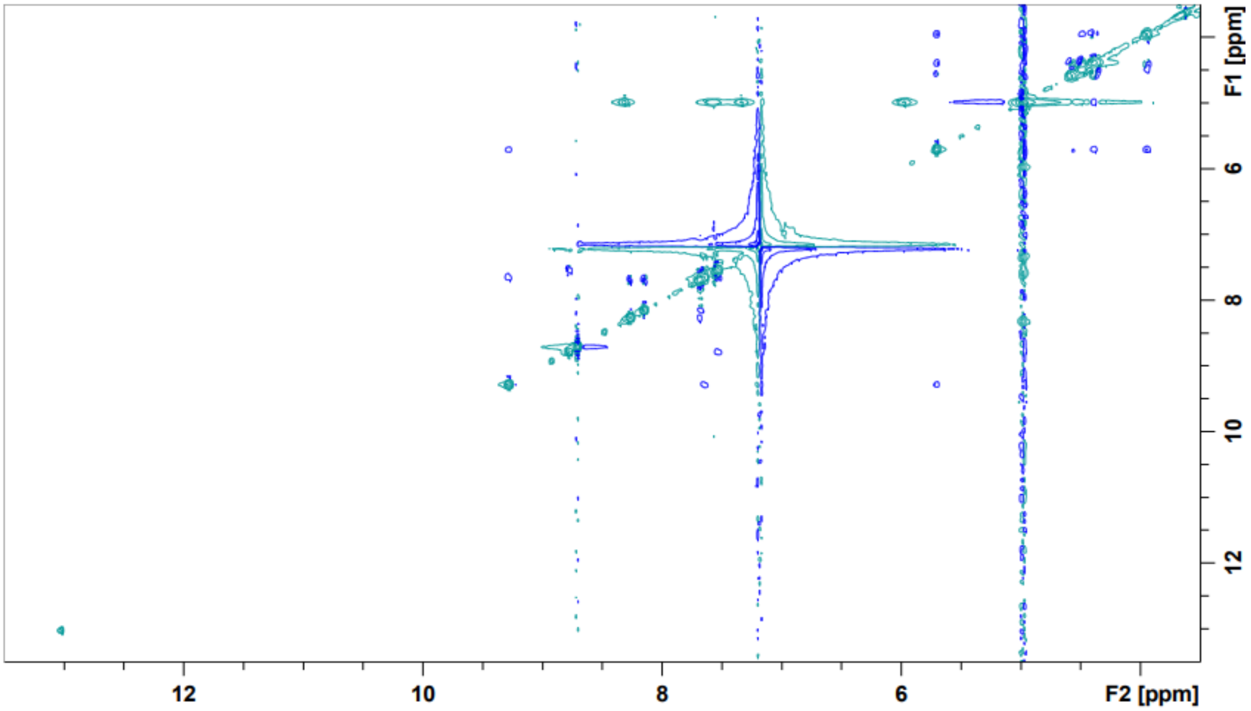

S8. IR of compound **1**

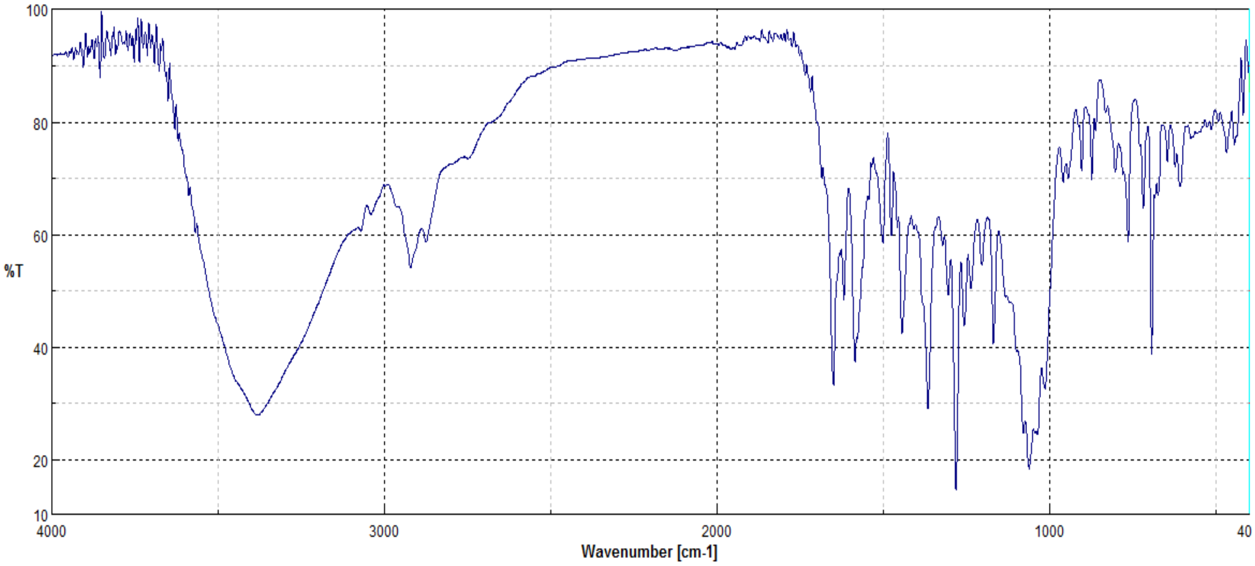

S9. MS of compound **1**

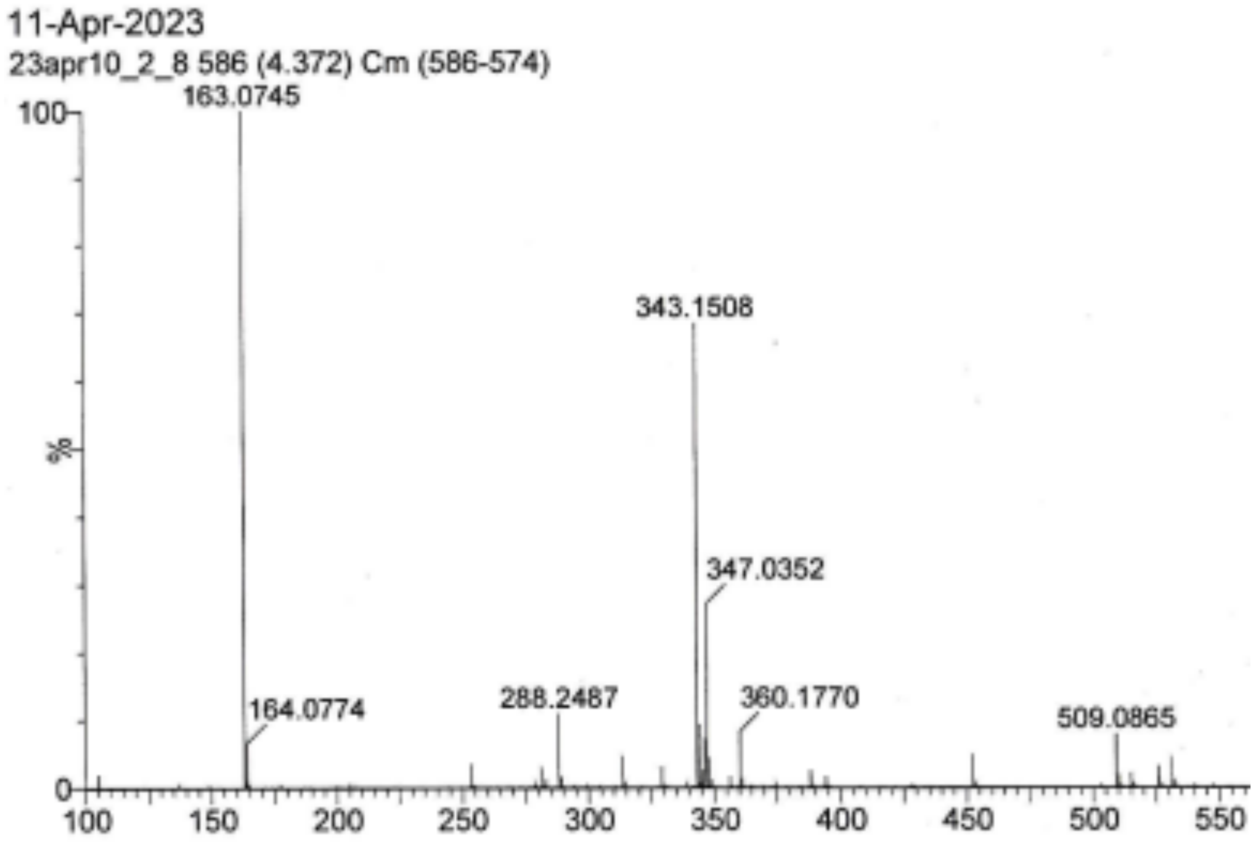

S10. MS of acetylated compound **1**

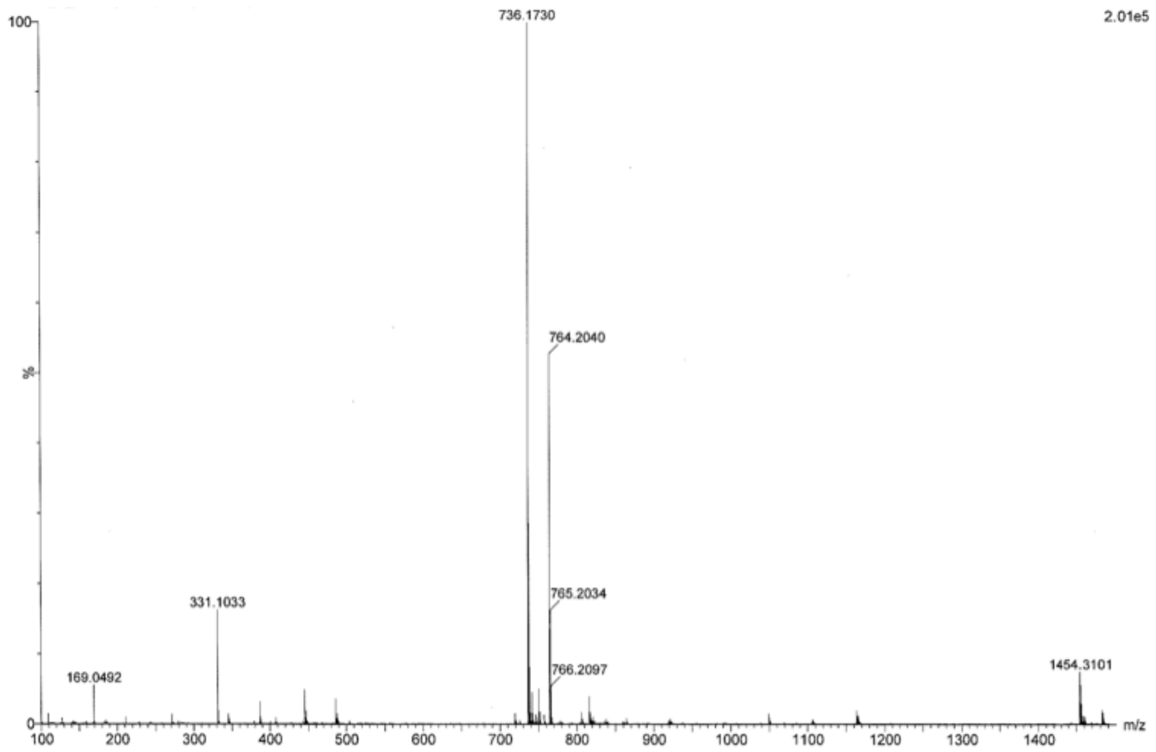

S11. Activity of compound **1**

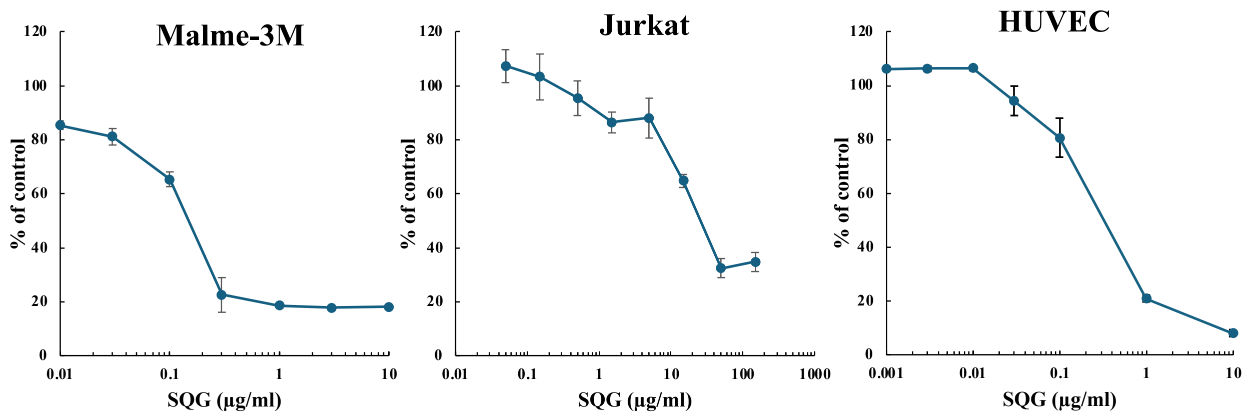

Supplement: Supplementary file 1 — Supporting Information [file 41429_2024_729_MOESM1_ESM.pdf]
